# A Mathematical Model of Cancer Treatment by Radiotherapy

**DOI:** 10.1155/2014/172923

**Published:** 2014-11-13

**Authors:** Zijian Liu, Chenxue Yang

**Affiliations:** ^1^School of Science, Chongqing Jiaotong University, Chongqing 400074, China; ^2^Department of Mathematics, Hangzhou Normal University, Hangzhou, Zhejiang 310036, China; ^3^School of Computer Science and Engineering, University of Electronic Science and Technology of China, Chengdu 610054, China

## Abstract

A periodic mathematical model of cancer treatment by radiotherapy is presented and studied in this paper. Conditions on the coexistence of the healthy and cancer cells are obtained. Furthermore, sufficient conditions on the existence and globally asymptotic stability of the positive periodic solution, the cancer eradication periodic solution, and the cancer win periodic solution are established. Some numerical examples are shown to verify the validity of the results. A discussion is presented for further study.

## 1. Introduction

Cancer is a well-known killer of humans worldwide, and its treatments are varied and sporadically successful. There are four main types of cancer treatments, which are surgery, chemotherapy, radiotherapy, and immunotherapy. In this paper, we only consider cancer treatment by radiotherapy.

Radiotherapy, as a primary treatment strategy, has been proven to be an effective tool in combating with cancer [1, 2]. Radiation therapy is a treatment procedure that uses radiation to kill malignant cells. This treatment targets rapidly reproducing cells such as those in cancer [3]. Therefore, when cancer cells are irradiated, there is a lesser effect on more slowly reproducing surrounding healthy cells. As such, the intent of this paper is to model the dynamics and interactions of healthy and cancer cells under radiation therapy.

It is an important and effective way to deeply understand the real-world problems by establishing mathematical models and analyzing their dynamical behaviors (see [4–9] and reference cited therein). Recently, some mathematical models that focus on cancer treatment by radiotherapy have been presented and studied ([10–13]). Liu et al. in [10] focused on dynamical behaviors of normal cells that affected periodic radiation and established some conditions on the permanence and extinction of the normal and radiated cells. Moreover, they obtained criteria on the existence and global asymptotic stability of unique positive periodic solutions of the system. Belostotski in [11] presented a mathematical model to represent the interactions between healthy and cancer cells subject to radiation, where the interactions between healthy and cancer cells were viewed as competition for bodily resources. He featured four different control mechanisms of radiation delivery. They included continuous constant radiation, continuous radiation that is proportional to the instantaneous cancer concentration, continuous radiation that is proportional to the ratio of cancer to healthy cell concentration, and periodic administration of radiation. He supposed that the effect of radiation on healthy cells ideally is zero and obtained some sufficient conditions on each case that guarantee the cancer to be cure or treatment. In paper [12], Belostotski and Freedman developed and analyzed a mathematical model of cancer treatment by radiotherapy using control theory, where the radioactivity only affected the cancer cells. Later, considering the fact that the radiation also may affect the healthy cells to some extent during the radiotherapy, Freedman and Belostotski in [13] extended the previous study by perturbing the previous models. They considered four types of treatment delivery: constant, linear, feedback, and perturbed periodic deliveries. For each case, they established some sufficient conditions on the cure state and treatment state. However, paper [13] only considered the perturbed periodic radiation, although paper [12] investigated the periodic radiation, it supposed that the effect of radiation on healthy cells is zero. Hence, the study of periodic radiation under conditions that both cancer and healthy cells are affected by radiation is of major importance.

This paper is organized as follows. In Section 2, we present our model and give a basic theorem. Conditions for the coexistence of the healthy cells and cancer cells of the system are obtained in Section 3. In Section 4, we establish some sufficient conditions on the existence and globally asymptotic stability of positive periodic solution, cancer eradication periodic solution, and cancer win periodic solution. Numerical simulations are shown to verify the validity of the theorems in Section 5. Finally, we discuss our results and present some interesting problems.

## 2. The Model

To simplify the model, we assume that the concentrations of cancer and healthy cells exist in the same region of the organism; the administration of radiation removes a large amount of cancer cells and a small amount of healthy cells from the system. Here, the terms “large” and “small” are used as a relation to the appropriate cell population at a particular location in the organism. Radiotherapy is in fact a control mechanism on the rates of change of the concentrations of cancer and healthy cells by harvesting them.

In a given tissue, let *x*_1_(*t*) be the concentration of healthy cells, and let *x*_2_(*t*) be the concentration of cancer cells; then our model takes the form

(1) $$\begin{array}{c} {\dot{x}}_{1}=\alpha_{1}x_{1}\left(1-\frac{x_{1}}{K_{1}}\right)-\beta_{1}x_{1}x_{2}-ɛD\left(t\right)x_{1},\\ {\dot{x}}_{2}=\alpha_{2}x_{2}\left(1-\frac{x_{2}}{K_{2}}\right)-\beta_{2}x_{1}x_{2}-D\left(t\right)x_{2}, \end{array}$$

where $\dot{x}=\text{d}x/\text{d}t$ and *D*(*t*) is the strategy of the radiotherapy. We assume that *D*(*t*) ≡ *γ* > 0 when *t* ∈ [*nω*, *nω* + *L*) (treatment stage) and *D*(*t*) ≡ 0 when *t* ∈ [*nω* + *L*, (*n* + 1)*ω*) (no treatment stage) for all *n* = 0,1, 2,…, where *ω* is the periodic of treatment and 0 < *L* < *ω* is the radiation treatment time. Further, in the absence of treatment (*D*(*t*) ≡ 0 for all *t*⩾0), the interactions between cancer and healthy cells were viewed as competition for bodily resources and the model was taken Lotka-Volterra competition type [14–16]. During the process of cancer radiation treatment, the healthy cells are also affected. The proportion of the radiation is *ɛD*(*t*), *ɛ* > 0 (*ɛ* = 0 is the ideal, but impossible to achieve in a practical scenario). *α*_*i*_ > 0  (*i* = 1,2) are the respective proliferation coefficients, *K*_*i*_  (*i* = 1,2) are the respective carrying capacities, and *β*_*i*_  (*i* = 1,2) are the respective competition coefficients.

In the absence of radiation, cancer (i.e., *x*_2_) wins resulting in the following conditions [14]:

(2) $$\begin{array}{c} K_{1}<\frac{\alpha_{2}}{\beta_{2}},\;\;K_{2}>\frac{\alpha_{1}}{\beta_{1}}. \end{array}$$

This yields one globally stable equilibrium at (*x*_1_, *x*_2_) = (0, *K*_2_) for positive initial values [14]. The following discussion of this paper will also base on the condition (2).

According to biological interpretation, we only consider the nonnegative solutions. Hence, we suppose that *x*_1_(0)⩾0 and *x*_2_(0)⩾0; then the following assertion is of major importance.

**Theorem 1 .**

(i) Nonnegative quadrant of *R* ^2^ is invariant for system (1). (ii) System (1) is ultimately bounded.

**Proof**

Let *x*_1_(*t*) and *x*_2_(*t*) be solutions of system (1). Here, we only analyze the healthy cells, the cancer cells can be analyzed similarly.

(i) If *x*_1_(*t*_0_) = 0 at some *t*_0_⩾0, then *x*_1_(*t*) ≡ 0 for all *t*⩾*t*_0_. If *x*_1_(*t*_0_) ≠ 0 for all *t*_0_⩾0, from the first equation of system (1),

(3) x1t=x10 ×exp⁡∫0tα11−x1sK1−β1x2s−ɛDsds⩾0,

for all *t*⩾0. Hence, *x*_1_(*t*)⩾0 for any nonnegative initial values.

(ii) From the first equation of system (1)

(4) $$\begin{array}{c} {\dot{x}}_{1}⩽\alpha_{1}x_{1}\left(1-\frac{x_{1}}{K_{1}}\right) \end{array}$$

for all *t*⩾0. According to the comparison theorem, there is a *T*_1_⩾0 such that *x*_1_(*t*) ⩽ *K*_1_ + 1 for all *t*⩾*T*_1_. *x*_1_(*t*) is ultimately bounded. This completes the proof.

## 3. Coexistence

In this section, we will investigate the coexistence of the healthy and cancer cells. We will find that when the radiation dosage *γ* is chosen from a given interval, the cancer cells will neither grow unrestricted nor tend to zero.

To understand the model more clearly, we rewrite system (1) as follows:

(5) x˙1=α1x11−x1K1−β1x1x2−ɛγx1x˙2=α2x21−x2K2−β2x1x2−γx2,t∈nω,nω+Ltreatment  stage,x˙1=α1x11−x1K1−β1x1x2x˙2=α2x21−x2K2−β2x1x2,t∈nω+L,n+1ωno  treatment  stage,n=0,1,2….

Before giving the main result of this section, we firstly consider the following two-specie Lotka-Volterra competitive system:

(6) $$\begin{array}{c} \dot{u}\left(t\right)=u\left(t\right)\left[b_{1}-a_{11}u\left(t\right)-a_{12}v\left(t\right)\right]\\ \dot{v}\left(t\right)=v\left(t\right)\left[b_{2}-a_{21}u\left(t\right)-a_{22}v\left(t\right)\right], \end{array}$$

where *b*_*i*_ > 0, *a*_*ij*_ > 0, *i*, *j* = 1,2. We have the following useful Lemma.

**Lemma 2 .**

System (6) has a unique positive equilibrium point (*u* ^*^, *v* ^*^) if inequality

(7) $$\begin{array}{c} \frac{a_{11}}{a_{21}}>\frac{b_{1}}{b_{2}}>\frac{a_{12}}{a_{22}} \end{array}$$

or

(8) $$\begin{array}{c} \frac{a_{12}}{a_{22}}>\frac{b_{1}}{b_{2}}>\frac{a_{11}}{a_{21}} \end{array}$$

is satisfied. Moreover, if inequality (7) is satisfied, then the equilibrium point (*u* ^*^, *v* ^*^) of system (6) is globally asymptotically stable and if inequality (8) is satisfied, (*u* ^*^, *v* ^*^) is unstable.

System (6) is a well-known Lotka-Volterra competitive system. The existence and stability of the positive equilibrium point have been studied in many articles and books, for example, [17]. Here we omit the proof of it.

On the coexistence of the healthy cells and cancer cells, we have the following theorem.

**Theorem 3 .**

Assume that the following conditions

(9) $$\begin{array}{c} \alpha_{2}-\gamma >0,\;\;\frac{\alpha_{1}}{K_{1}\beta_{2}}>\frac{\alpha_{1}-ɛ\gamma }{\alpha_{2}-\gamma }>\frac{K_{2}\beta_{1}}{\alpha_{2}} \end{array}$$

hold. Then the healthy cells and cancer cells are coexistent.

**Proof**

From system (5) we have

(10) $$\begin{array}{c} {\dot{x}}_{1}\left(t\right)⩾x_{1}\left(t\right)\left[\alpha_{1}-ɛ\gamma -\frac{\alpha_{1}}{K_{1}}x_{1}\left(t\right)-\beta_{1}x_{2}\left(t\right)\right]\\ {\dot{x}}_{2}\left(t\right)⩾x_{2}\left(t\right)\left[\alpha_{2}-\gamma -\beta_{2}x_{1}\left(t\right)-\frac{\alpha_{2}}{K_{2}}x_{2}\left(t\right)\right] \end{array}$$

for all *t*⩾0. Using the comparison theorem (see [18, 19]) we have *x*_1_(*t*)⩾*u*(*t*) and *x*_2_(*t*)⩾*v*(*t*) for all *t*⩾0, where (*u*(*t*), *v*(*t*)) is the solution of the following auxiliary system:

(11) $$\begin{array}{c} \dot{u}\left(t\right)=u\left(t\right)\left[\alpha_{1}-ɛ\gamma -\frac{\alpha_{1}}{K_{1}}u\left(t\right)-\beta_{1}v\left(t\right)\right]\\ \dot{v}\left(t\right)=v\left(t\right)\left[\alpha_{2}-\gamma -\beta_{2}u\left(t\right)-\frac{\alpha_{2}}{K_{2}}v\left(t\right)\right] \end{array}$$

with the initial values *u*(0) = *x*_1_(0) and *v*(0) = *x*_2_(0). By condition (9) and Lemma 2, we know that system (11) has a unique positive equilibrium point (*u* ^*^, *v* ^*^) which is globally asymptotically stable. Hence, there is a *T*_2_ > 0 such that *x*_1_(*t*)⩾*u*(*t*)⩾*u* ^*^/2 > 0 and *x*_2_(*t*)⩾*v*(*t*)⩾*v* ^*^/2 > 0 for all *t*⩾*T*_2_, which imply that system (1) is permanent; that is, the healthy cells and cancer cells are coexistent. This completes the proof.

**Remark 4 .**

Condition (9) implies the range of radiation dosage *γ*. In fact, from condition (9), on the one hand we have *γ* < *α*_1_/*ɛ* and *γ* < *α*_2_; on the other hand we have

(12) $$\begin{array}{c} \left(\alpha_{1}-ɛK_{1}\beta_{2}\right)\gamma <\alpha_{1}\alpha_{2}-K_{1}\beta_{2}\alpha_{1},\\ \left(ɛ\alpha_{2}-K_{2}\beta_{1}\right)\gamma <\alpha_{1}\alpha_{2}-K_{2}\beta_{1}\alpha_{2}. \end{array}$$

Combine the above two inequalities; there is

(13) α1−K2β1+ɛα2−K1β2γ  <α2α1−K2β1+α1α2−K1β2.

However, we cannot judge the sign of the coefficient of *γ* in inequality (13) only by the conditions (2) and (9). This leads to three cases.

(a) Consider (*α*_1_ − *K*_2_ *β*_1_) + *ɛ*(*α*_2_ − *K*_1_ *β*_2_) > 0. If *α*_2_(*α*_1_ − *K*_2_ *β*_1_) + *α*_1_(*α*_2_ − *K*_1_ *β*_2_) > 0, then

(14) $$\begin{array}{c} 0<\gamma <\min\left\{\frac{\alpha_{2}\left(\alpha_{1}-K_{2}\beta_{1}\right)+\alpha_{1}\left(\alpha_{2}-K_{1}\beta_{2}\right)}{\left(\alpha_{1}-K_{2}\beta_{1}\right)+ɛ\left(\alpha_{2}-K_{1}\beta_{2}\right)},\frac{\alpha_{1}}{ɛ},\alpha_{2}\right\}. \end{array}$$

If *α*_2_(*α*_1_ − *K*_2_ *β*_1_) + *α*_1_(*α*_2_ − *K*_1_ *β*_2_) ⩽ 0, *γ* is inexistent.

(b) Consider (*α*_1_ − *K*_2_ *β*_1_) + *ɛ*(*α*_2_ − *K*_1_ *β*_2_) = 0. If *α*_2_(*α*_1_ − *K*_2_ *β*_1_) + *α*_1_(*α*_2_ − *K*_1_ *β*_2_) > 0, then 0 < *γ* < min⁡{*α*_1_/*ɛ*, *α*_2_}. If *α*_2_(*α*_1_ − *K*_2_ *β*_1_) + *α*_1_(*α*_2_ − *K*_1_ *β*_2_) ⩽ 0, *γ* is inexistent.

(c) Consider (*α*_1_ − *K*_2_ *β*_1_) + *ɛ*(*α*_2_ − *K*_1_ *β*_2_) < 0. If *α*_2_(*α*_1_ − *K*_2_ *β*_1_) + *α*_1_(*α*_2_ − *K*_1_ *β*_2_)⩾0, then 0 < *γ* < min⁡{*α*_1_/*ɛ*, *α*_2_}. If *α*_2_(*α*_1_ − *K*_2_ *β*_1_) + *α*_1_(*α*_2_ − *K*_1_ *β*_2_) < 0, then *γ* > [*α*_2_(*α*_1_ − *K*_2_ *β*_1_) + *α*_1_(*α*_2_ − *K*_1_ *β*_2_)]/[(*α*_1_ − *K*_2_ *β*_1_) + *ɛ*(*α*_2_ − *K*_1_ *β*_2_)]. Furthermore, if [*α*_2_(*α*_1_ − *K*_2_ *β*_1_) + *α*_1_(*α*_2_ − *K*_1_ *β*_2_)]/[(*α*_1_ − *K*_2_ *β*_1_) + *ɛ*(*α*_2_ − *K*_1_ *β*_2_)] < min⁡{*α*_1_/*ɛ*, *α*_2_}, then

(15) $$\begin{array}{c} \left\{\frac{\alpha_{2}\left(\alpha_{1}-K_{2}\beta_{1}\right)+\alpha_{1}\left(\alpha_{2}-K_{1}\beta_{2}\right)}{\left(\alpha_{1}-K_{2}\beta_{1}\right)+ɛ\left(\alpha_{2}-K_{1}\beta_{2}\right)}\right\}<\gamma <\min\left\{\frac{\alpha_{1}}{ɛ},\alpha_{2}\right\}. \end{array}$$

If [*α*_2_(*α*_1_ − *K*_2_ *β*_1_) + *α*_1_(*α*_2_ − *K*_1_ *β*_2_)]/[(*α*_1_ − *K*_2_ *β*_1_) + *ɛ*(*α*_2_ − *K*_1_ *β*_2_)]⩾min⁡{*α*_1_/*ɛ*, *α*_2_}, *γ* is inexistent.

**Remark 5 .**

Condition (9) guarantees that the comparison system (11) has a globally asymptotically stable equilibrium point. Therefore, we can obtain that system (1) has a positive lower bound. However, if the condition

(16) $$\begin{array}{c} \alpha_{2}-\gamma >0,\;\;\frac{\alpha_{1}}{K_{1}\beta_{2}}<\frac{\alpha_{1}-ɛ\gamma }{\alpha_{2}-\gamma }<\frac{K_{2}\beta_{1}}{\alpha_{2}} \end{array}$$

is satisfied, that is, the positive equilibrium point of the comparison system (11) is a saddle point. How to choose the radiation dosage *γ* and what dynamical behavior of system (1) are still open problems.

**Remark 6 .**

Under conditions of Theorem 3, system (1) has a positive *ω*-periodic solution. The biological meanings can be understood as follows. If there is no treatment, from condition (2) we know that system (1) has a globally stable equilibrium point (0, *K*_2_) for any positive initial values. However, if we treat it all the time, by condition (9) and Lemma 2, we know that system (11) has a unique globally asymptotically stable positive equilibrium point (*u* ^*^, *v* ^*^). Hence, if the treatment is periodic, the solution of system (1) will tend to the positive equilibrium point (*u* ^*^, *v* ^*^) during the treatment stage, and it will tend to the cancer win state (0, *K*_2_) during the no treatment stage. This will lead to the appearance of a periodic solution.

## 4. Periodic Solutions

The existence of a positive *ω*-periodic solution is guaranteed by Remark 6. In the following, we will firstly give some criteria on the existence of cancer eradication periodic solution and cancer win periodic solution of system (1). Whereafter, we will establish some conditions under which each periodic solution is globally asymptotically stable.

Firstly, let us investigate the existence of cancer eradication periodic solution of the system. Consider the following subsystem of system (1) under the case that *x*_2_(*t*) ≡ 0:

(17) x˙1=α1x11−x1K1−ɛγx1, t∈nω,nω+L,x˙1=α1x11−x1K1, t∈nω+L,n+1ω,n=0,1,2….

When *t* ∈ [0, *L*), we have

(18) x1t=α1K1α1−ɛγ  +1x10−α1K1α1−ɛγexp⁡−α1−ɛγt−1,

from the continuity of *x*_1_(*t*),

(19) x1L=α1K1α1−ɛγ +1x10−α1K1α1−ɛγexp⁡−α1−ɛγL−1.

Then when *t* ∈ [*L*, *ω*), we have

(20) $$\begin{array}{c} x_{1}\left(t\right)={\left[\frac{1}{K_{1}}+\left(\frac{1}{x_{1}\left(L\right)}-\frac{1}{K_{1}}\right)\exp\left\{-\alpha_{1}\left(t-L\right)\right\}\right]}^{-1}; \end{array}$$

hence,

(21) x1ω=1K1+1x1L−1K1exp⁡−α1ω−L−1=1K1+α1K1α1−ɛγ−1K1     +1x10−α1K1α1−ɛγ     ×exp⁡−α1−ɛγL  ×exp⁡⁡−α1ω−L−1.

If system (17) has a positive *ω*-periodic solution, then we need *x*_1_(*ω*) = *x*_1_(0), which leads to

(22) x10=1−exp⁡ɛγL−α1ω ×1K1+α1K1α1−ɛγ−1K1exp⁡−α1ω−L   −α1K1α1−ɛγexp⁡⁡ɛγL−α1ω−1.

From expression (22) we can calculate that *x*_1_(0) > 0 is equivalent to *ɛγL* < *α*_1_ *ω*. Then we get the following result.

**Theorem 7 .**

System (1) has a cancer eradication periodic solution (*x*_1_^*^(*t*), 0) if the inequality *ɛγL* < *α*_1_ *ω* is satisfied.

The existence of the cancer win periodic solution of system (1) can be analyzed similarly. Here we only show the result.

**Theorem 8 .**

System (1) has a cancer win periodic solution (0, *x*_2_^*^(*t*)) if the inequality *γL* < *α*_2_ *ω* is satisfied.

Up to now, we have completed the studies on the existences of the positive periodic solution, the cancer eradication periodic solution, and the cancer win periodic solution of system (1). In the following of this section, we will mainly study the global stability on each periodic solution.

Let (*x*_1_^*^(*t*), *x*_2_^*^(*t*)) be a positive periodic solution of system (1). Choose

(23) $$\begin{array}{c} V\left(t\right)=\left|\ln x_{1}\left(t\right)-\ln x_{1}^{*}\left(t\right)\right|+\left|\ln x_{2}\left(t\right)-\ln x_{2}^{*}\left(t\right)\right|, \end{array}$$

where (*x*_1_(*t*), *x*_2_(*t*)) is any solution of system (1). Calculating the derivative of *V*(*t*) along system (5), when *t* ∈ [*nω*, *nω* + *L*), we have

(24) V˙t=sgn⁡x1−x1∗ ×α1−ɛγ−α1K1x1−β1x2   −α1−ɛγ−α1K1x1∗−β1x2∗ +sgn⁡x2−x2∗ ×α2−γ−α2K2x2−β2x1   −α2−γ−α2K2x2∗−β2x1∗=−α1K1x1−x1∗−α2K2x2−x2∗ +sgn⁡x1−x1∗−β1x2−x2∗ +sgn⁡x2−x2∗−β2x1−x1∗⩽−α1K1−β2x1−x1∗−α2K2−β1x2−x2∗.

When *t* ∈ [*nω* + *L*, (*n* + 1)*ω*), we get

(25) V˙t=sgn⁡x1−x1∗ ×α1−α1K1x1−β1x2−α1−α1K1x1∗−β1x2∗ +sgn⁡x2−x2∗ ×α2−α2K2x2−β2x1−α2−α2K2x2∗−β2x1∗⩽−α1K1−β2x1−x1∗−α2K2−β1x2−x2∗.

Consequently, if *α*_1_ > *K*_1_ *β*_2_ and *α*_2_ > *K*_2_ *β*_1_, from (24) and (25), we have $\dot{V}(t)<0$ for all *t*⩾0. By Lyapunov stability theory (see [18, 19]), the following theorem is obtained immediately.

**Theorem 9 .**

The positive periodic solution (*x*_1_^*^(*t*), *x*_2_^*^(*t*)) of system (1) is unique and globally asymptotically stable if the conditions *α*_1_ > *K*_1_ *β*_2_ and *α*_2_ > *K*_2_ *β*_1_ are satisfied.

On the uniqueness and global stabilities of the cancer eradication periodic solution and cancer win periodic solution, we have the following results.

**Theorem 10 .**

Assume that condition *ɛγL* < *α*_1_ *ω* holds. Further, if

(26) $$\begin{array}{c} \beta_{1}<\beta_{2},\;\;\alpha_{1}-ɛ\gamma -\frac{\alpha_{1}}{K_{1}}\sigma_{1}<0,\\ \alpha_{2}-\gamma <0,\;\;\gamma \left(\omega -L\right)<\eta \omega \end{array}$$

are satisfied, where *σ*_1_ = min⁡_*t*∈[0,*ω*]_{*x*_1_^*^(*t*)} and −*η* = max⁡{*α*_1_ − *ɛγ* − *α*_1_ *σ*_1_/*K*_1_, *α*_2_ − *γ*}, then system (1) has a unique globally asymptotically stable cancer eradication periodic solution.

**Proof**

The existence of the cancer eradication periodic solution has been established by Theorem 7; then we will mainly prove its uniqueness and global stability.

Let (*x*_1_(*t*), *x*_2_(*t*)) be any solution of system (1). Choose

(27) $$\begin{array}{c} V\left(t\right)=\left|x_{1}\left(t\right)-x_{1}^{*}\left(t\right)\right|+x_{2}\left(t\right). \end{array}$$

When *t* ∈ [*nω*, *nω* + *L*), by condition (26) and calculating the derivative of *V*(*t*) along system (5), we have

(28) V˙t=sgn⁡x1−x1∗ ×α1x1−ɛγx1−α1K1x12−β1x1x2   −α1x1∗−ɛγx1∗−α1K1x1∗2 +α2x2−γx2−α2K2x22−β2x1x2⩽α1−ɛγx1−x1∗−α1K1x1+x1∗x1−x1∗ +β1−β2x1x2+α2−γx2⩽α1−ɛγ−α1K1σ1x1−x1∗+α2−γx2⩽−ηVt.

Hence, *V*(*t*) ⩽ *V*(*nω*)exp⁡{−*η*(*t* − *nω*)} ⩽ *V*(*nω*) for all *t* ∈ [*nω*, *nω* + *L*). According to the continuity of the solution, especially, we have *V*(*nω* + *L*) ⩽ *V*(*nω*).

However, when *t* ∈ [*nω* + *L*, (*n* + 1)*ω*), by condition (26) and calculating the derivative of *V*(*t*) along system (5), we have

(29) V˙t=sgn⁡x1−x1∗ ×α1x1−α1K1x12−β1x1x2−α1x1∗−α1K1x1∗2 +α2x2−α2K2x22−β2x1x2⩽α1−α1K1σ1x1−x1∗+α2x2⩽γ−ηVt.

Consequently, *V*(*t*) ⩽ *V*(*nω* + *L*)exp⁡{(*γ* − *η*)(*t* − *nω* − *L*)} ⩽ *MV*(*nω*) for all *t* ∈ [*nω* + *L*, (*n* + 1)*ω*), where *M* = exp⁡{|*γ* − *η* | (*ω* − *L*)}⩾1 and *M* is bounded. From the above analysis, we know that

(30) $$\begin{array}{c} V\left(t\right)⩽MV\left(n\omega \right) \end{array}$$

for all *t* ∈ [*nω*, (*n* + 1)*ω*).

By a simple calculation and from the last inequality of condition (26), it can be obtained that *V*(*nω*) ⩽ *V*(0)exp⁡{*n*[*γ*(*ω* − *L*) − *ηω*]} → 0 as *n* → *∞*. Together with (30), we finally have *V*(*t*) → 0 as *t* → *∞*. Hence, *x*_1_(*t*) → *x*_1_^*^(*t*) and *x*_2_(*t*) → 0 as *t* → *∞*. The cancer eradication periodic solution is unique and global stable. This completes the proof.

**Theorem 11 .**

Assume that condition *γL* < *α*_2_ *ω* holds. Further, if

(31) $$\begin{array}{c} \alpha_{1}>K_{1}\beta_{2},\;\;\alpha_{2}>K_{2}\beta_{1},\;\;\alpha_{1}+\beta_{2}-\beta_{1}\sigma_{2}<0 \end{array}$$

are satisfied, where *σ*_2_ = min⁡_*t*∈[0,*ω*]_{*x*_2_^*^(*t*)}. Then system (1) has a unique globally asymptotically stable cancer win periodic solution.

**Proof**

Let (*x*_1_(*t*), *x*_2_(*t*)) be any solution of system (1). Choose

(32) $$\begin{array}{c} V\left(t\right)=x_{1}\left(t\right)+\left|\ln x_{2}\left(t\right)-\ln x_{2}^{*}\left(t\right)\right|. \end{array}$$

Proceed the similar analysis as Theorem 10; condition (31) can be obtained easily. Here we omit the proof of it.

**Remark 12 .**

In the discussion of the existence and global stability of the positive periodic solution, we need the conditions that *γ* < *α*_1_/*ɛ* and *γ* < *α*_2_. However, from the fact *L* < *ω* we realize that *γ* > *α*_1_/*ɛ* is allowed in the existence and global stability of the cancer eradication periodic solution by Theorems 7 and 10 and *γ* > *α*_2_ is also allowed in the existence and global stability of the cancer win periodic solution by Theorems 8 and 11. These show that a high radiation dosage may kill the cancer cells all (Theorem 10) and it may also kill the healthy cells all (Theorem 11).

## 5. Numerical Illustrations

**Figure 1:**
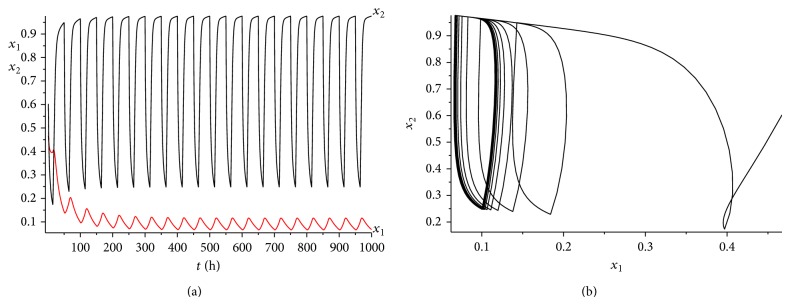
The dynamics of system (1) with *L* = 15. (a) The time series for healthy cells *x*_1_ and cancer cells *x*_2_. (b) The phase of healthy cells *x*_1_ and cancer cells *x*_2_. Obviously, system (1) has a globally stable positive 50-periodic solution.

**Figure 2:**
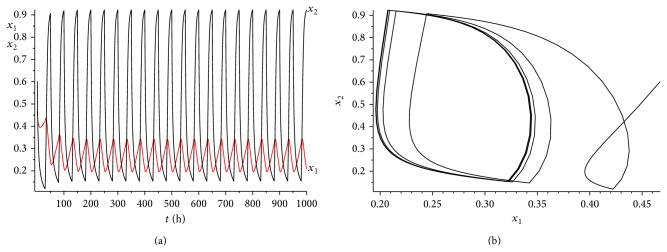
The dynamics of system (1) with *L* = 30. (a) The time series for healthy cells *x*_1_ and cancer cells *x*_2_. (b) The phase of healthy cells *x*_1_ and cancer cells *x*_2_. Obviously, system (1) has a globally stable positive 50-periodic solution.

**Figure 3:**
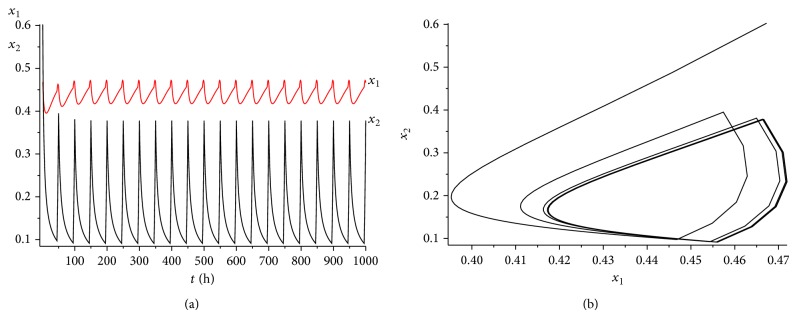
The dynamics of system (1) with *L* = 45. (a) The time series for healthy cells *x*_1_ and cancer cells *x*_2_. (b) The phase of healthy cells *x*_1_ and cancer cells *x*_2_. Obviously, system (1) has a globally stable positive 50-periodic solution.

In this section, we give three groups of numerical examples to verify the validity of the three cases of periodic solutions, respectively. We consider system (1) with the following coefficients in Theorem 9: *α*_1_ = 0.1, *α*_2_ = 0.45, *β*_1_ = 0.11, *β*_2_ = 0.15, *K*_1_ = 0.65, and *K*_2_ = 1. Obviously, condition (2) is satisfied. If the rate to the healthy cells from the radiation is chosen as *ɛ* = 0.05, then we have *μ*_1_ = (*α*_1_ − *K*_2_ *β*_1_) + *ɛ*(*α*_2_ − *K*_1_ *β*_2_) = 0.007625 > 0 and *μ*_2_ = *α*_2_(*α*_1_ − *K*_2_ *β*_1_) + *α*_1_(*α*_2_ − *K*_1_ *β*_2_) = 0.03075 > 0. The range of *γ* is determined by case (a) in Remark 4. Calculating it simply, we get that 0 < *γ* < 0.45. We choose *γ* = 0.35 here. It is easy to verity that all the conditions of Theorems 3 and 9 are satisfied. Hence, when we consider 50 hours as a treatment period, system (1) has a unique globally asymptotically stable positive 50-periodic solution. See Figures 1–3. The treatment time is chosen as *L* = 15 hours (Figure 1), *L* = 30 hours (Figure 2), and *L* = 45 hours (Figure 3), respectively.

From Figures 1–3, it is interesting to observe that as the increasing of the treatment time in a periodic treatment, the concentration of the healthy cells will increase and the concentration of the cancer cells will decrease. But we cannot eradicate the cancer cells no matter how to choose the treatment time *L* in this situation.

**Figure 4:**
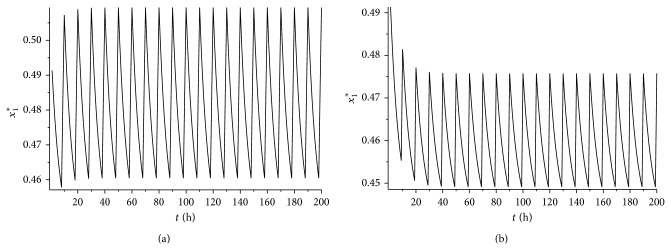
(a) The time series for the cancer eradication periodic solution *x*_1_^*^(*t*) with *L* = 8 and *γ* = 0.65. (b) The time series for the cancer eradication periodic solution *x*_1_^*^(*t*) with *L* = 9 and *γ* = 0.65.

**Figure 5:**
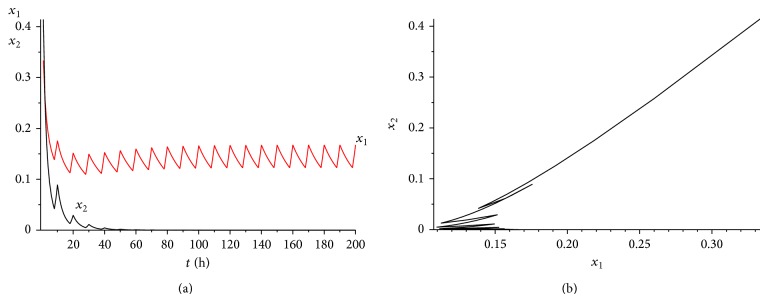
The dynamics of system (1) with *L* = 8 and *γ* = 0.65. (a) The time series for healthy cells *x*_1_ and cancer cells *x*_2_. (b) The phase of healthy cells *x*_1_ and cancer cells *x*_2_. Obviously, system (1) has a globally stable cancer eradication 10-periodic solution.

**Figure 6:**
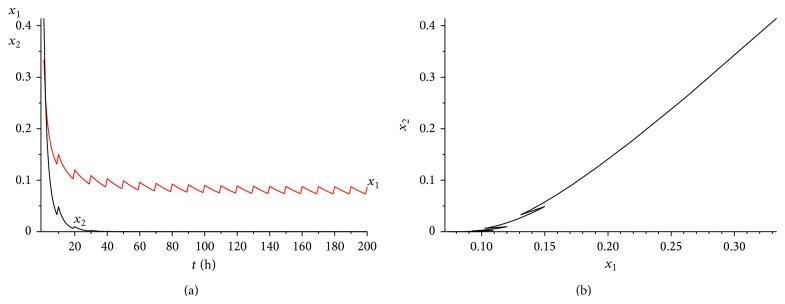
The dynamics of system (1) with *L* = 9 and *γ* = 0.65. (a) The time series for healthy cells *x*_1_ and cancer cells *x*_2_. (b) The phase of healthy cells *x*_1_ and cancer cells *x*_2_. Obviously, system (1) has a globally stable cancer eradication 10-periodic solution.

To show the existence and global stability of the cancer eradication periodic solution, we illustrate Theorem 10 with the coefficients in system (1) as *α*_1_ = 0.2, *α*_2_ = 0.5, *β*_1_ = 0.5, *β*_2_ = 0.55, *K*_1_ = 0.65, *K*_2_ = 1. Then, *α*_2_/*β*_2_ − *K*_1_ = 0.2591 > 0 and *α*_1_/*β*_1_ − *K*_2_ = −0.6000 < 0, condition (2) is satisfied. We choose *γ* = 0.65 and *ɛ* = 0.3. Obviously, if we choose the periodic of treatment *ω* = 10 hours, by the fact *L* < *ω*, we have *ɛγL* < *α*_1_ *ω*. The cancer eradication periodic solution (*x*_1_^*^(*t*), 0) exists by Theorem 7. See Figure 4(a) (*L* = 8 hours) and Figure 4(b) (*L* = 9 hours). From Figure 4(a), we can obtain that *σ*_1_ = min⁡_*t*∈[0,*ω*]_{*x*_1_^*^(*t*)}⩾0.44, and, Figure 4(b), *σ*_1_⩾0.44. It is easy to verify that condition (26) is satisfied. According to Theorem 10, system (1) has a unique globally asymptotically stable cancer eradication 10-periodic solution. See Figures 5(a) and 5(b) (*L* = 8 hours) and Figures 6(a) and 6(b) (*L* = 9 hours).

It can be seen from Figures 5(a) and 5(b) and Figures 6(a) and 6(b) that if all the other conditions are not changed, the increase of the treatment time in a periodic treatment will quicken the extinction of the cancer cells and decrease the concentration of the healthy cells at the same time.

As we all know, larger dosage radiation can kill cancer cells more effectively, but it also may increase the rate to the healthy cells from the radiation. We now investigate effects of the variance of the parameter *γ* to the cancer eradication periodic solution with *ω* = 10 hours and *L* = 8 hours. Here, we also choose *ɛ* = 0.3. For the sake of getting a cancer eradication periodic solution, we need the condition *ɛγL* < *α*_1_ *ω*. Then we have *γ* < *α*_1_ *ω*/(*ɛL*) = 0.83. However, the true Theorem 10 needs condition (26), which includes that *γ*(*ω* − *L*)+(−*η*)*ω* < 0. From the fact that −*η* = max⁡{*α*_1_ − *ɛγ* − *α*_1_ *σ*_1_/*K*_1_, *α*_2_ − *γ*}, then we have *γ*(*ω* − *L*)+(*α*_2_ − *γ*)*ω* < *γ*(*ω* − *L*)+(−*η*)*ω* < 0, which implies *γ* > 0.625. Hence, here we choose *γ* = 0.65,0.75 and 0.8 separately to investigate the influence of the variance of the *γ* to the cancer eradication periodic solution. The dynamical behavior of the system with *γ* = 0.65 can be seen in Figures 5(a) and 5(b).

**Figure 7:**
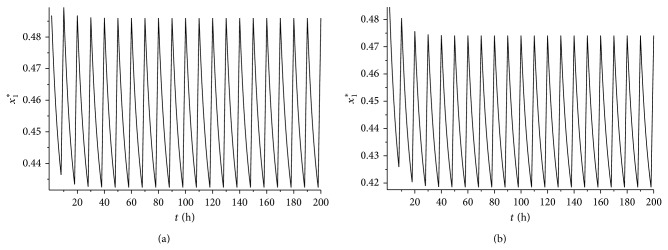
(a) The time series for the cancer eradication periodic solution *x*_1_^*^(*t*) with *L* = 8 and *γ* = 0.75. (b) The time series for the cancer eradication periodic solution *x*_1_^*^(*t*) with *L* = 8 and *γ* = 0.8.

**Figure 8:**
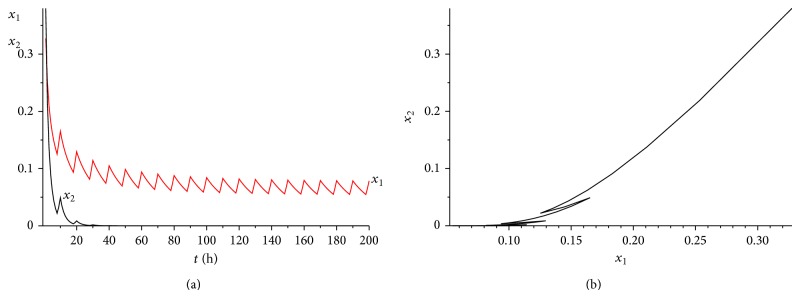
The dynamics of system (1) with *L* = 8 and *γ* = 0.75. (a) The time series for healthy cells *x*_1_ and cancer cells *x*_2_. (b) The phase of healthy cells *x*_1_ and cancer cells *x*_2_. Obviously, system (1) has a globally stable cancer eradication 10-periodic solution.

**Figure 9:**
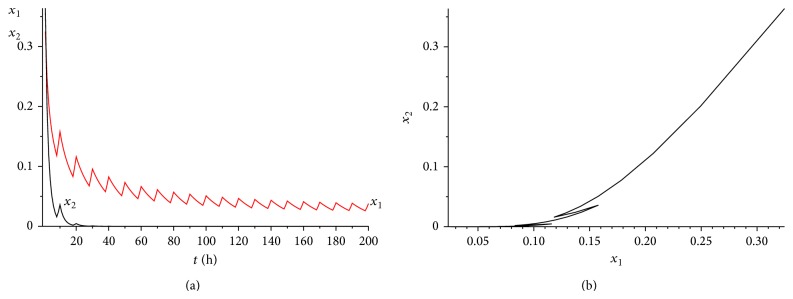
The dynamics of system (1) with *L* = 8 and *γ* = 0.8. (a) The time series for healthy cells *x*_1_ and cancer cells *x*_2_. (b) The phase of healthy cells *x*_1_ and cancer cells *x*_2_. Obviously, system (1) has a globally stable cancer eradication 10-periodic solution.

If we take *γ* = 0.75, it is easy to verify that *ɛγL* < *α*_1_ *ω* and system (1) has a cancer eradication periodic solution. See Figure 7(a). From Figure 7(a) we obtain *σ*_1_ = min⁡_*t*∈[0,*ω*]_{*x*_1_^*^(*t*)}⩾0.43; then condition (26) is satisfied. According to Theorem 10, system (1) has a unique globally asymptotically stable cancer eradication 10-periodic solution. See Figures 8(a) and 8(b). If *γ* is taken as 0.8, condition *ɛγL* < *α*_1_ *ω* is also satisfied, then system (1) has a cancer eradication periodic solution. See Figure 7(b). From Figure 7(b) we obtain *σ*_1_ = min⁡_*t*∈[0,*ω*]_{*x*_1_^*^(*t*)}⩾0.42, then it is easy to verify that condition (26) is satisfied. According to Theorem 10, system (1) has a unique globally asymptotically stable cancer eradication 10-periodic solution. See Figures 9(a) and 9(b). It can be seen from Figures 5(a) and 5(b) (*γ* = 0.65), Figures 8(a) and 8(b) (*γ* = 0.75), and Figures 9(a) and 9(b) (*γ* = 0.8) that under the circumstance that the system has a cancer eradication periodic solution and all the other conditions are not changed, the increase of the radiation dosage will quicken the extinction of the cancer cells but will also decrease the concentration of the healthy cells at the same time. One can refer to the three figures; in Figure 5 with *γ* = 0.65, 0.1 ⩽ *x*_1_, in Figure 8 with *γ* = 0.75, 0.05 ⩽ *x*_1_ ⩽ 0.1, and in Figure 9 with *γ* = 0.8, *x*_1_ ⩽ 0.05.

**Figure 10:**
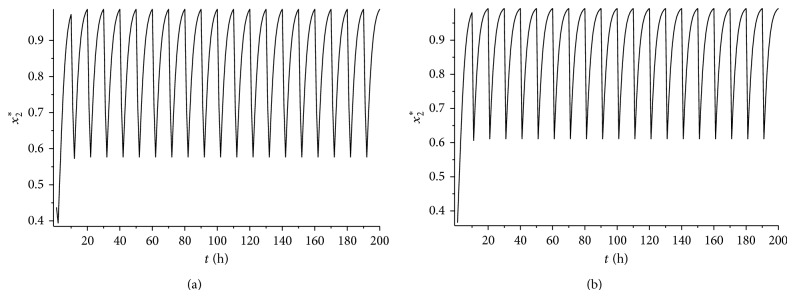
(a) The time series for the cancer win periodic solution *x*_2_^*^(*t*) with *γ* = 0.4 and *L* = 2. (b) The time series for the cancer win periodic solution *x*_2_^*^(*t*) with *γ* = 0.6 and *L* = 1.

**Figure 11:**
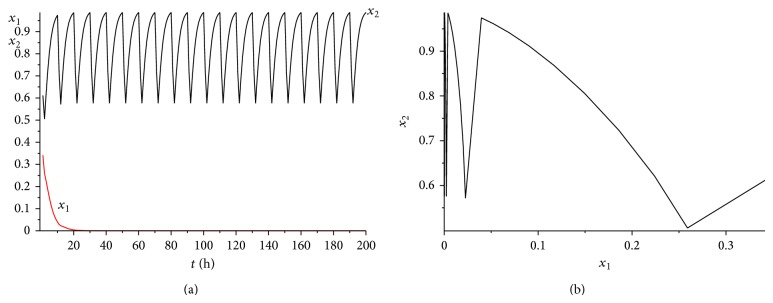
The dynamics of system (1) with *γ* = 0.4 and *L* = 2. (a) The time series for healthy cells *x*_1_ and cancer cells *x*_2_. (b) The phase of healthy cells *x*_1_ and cancer cells *x*_2_. Obviously, system (1) has a globally stable cancer win 10-periodic solution.

**Figure 12:**
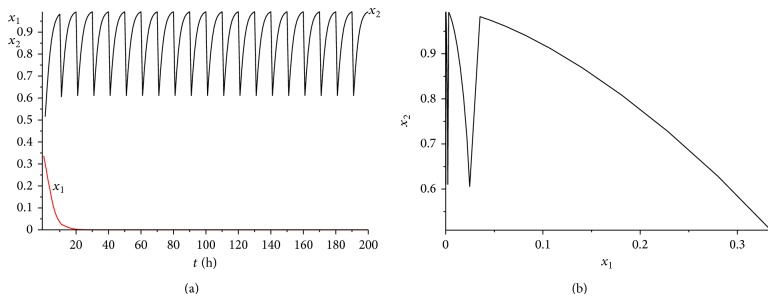
The dynamics of system (1) with *γ* = 0.6 and *L* = 1. (a) The time series for healthy cells *x*_1_ and cancer cells *x*_2_. (b) The phase of healthy cells *x*_1_ and cancer cells *x*_2_. Obviously, system (1) has a globally stable cancer win 10-periodic solution.

It is also a helpful suggestion for doctors that under what situation the cancer will win the competition. Theorem 11 gives us a useful result. Let us illustrate the theorem more clearly applying the following numerical examples. Choose the coefficients *α*_1_ = 0.2, *α*_2_ = 0.5, *β*_1_ = 0.48, *β*_2_ = 0.05, *K*_1_ = 0.65, and *K*_2_ = 1. It is easy to verify that condition (2) is satisfied. We choose the periodic of treatment *ω* = 10 hours and the rate to the healthy cells from the radiation is *ɛ* = 0.3. Firstly, we let the radiation dosage *γ* = 0.4 < *α*_2_. From the fact that *L* < *ω* we know that *γL* < *α*_2_ *ω*, then system (1) has a cancer win periodic solution (0, *x*_2_^*^(*t*)) by Theorem 8. See Figure 10(a), where the treatment time *L* = 2 hours. From Figure 10(a), we can obtain that *σ*_2_ = min⁡_*t*∈[0,*ω*]_{*x*_2_^*^(*t*)}⩾0.55. It is easy to verify that condition (31) is satisfied. According to Theorem 11, system (1) has a unique globally asymptotically stable cancer win 10-periodic solution. See Figures 11(a) and 11(b). However, by Remark 12, we know that the radiation dosage *γ* > *α*_2_ is also permitted. Hence, we choose *γ* = 0.6 > *α*_2_ in Figure 12. Then we have *γL* < *α*_2_ *ω*, system (1) has a cancer win periodic solution (0, *x*_2_^*^(*t*)) by Theorem 8. See Figure 10(b), where the treatment time *L* = 1 hour. From Figure 10(b), we can obtain that *σ*_2_ = min⁡_*t*∈[0,*ω*]_{*x*_2_^*^(*t*)}⩾0.6. It is easy to verify that condition (31) is satisfied. According to Theorem 11, system (1) has a unique globally asymptotically stable cancer win 10-periodic solution. See Figures 12(a) and 2(b). It can be seen from Figures 11(a) and 11(b) and Figures 12(a) and 12(b) that the cancer treatment is affected by both the radiation dosage and the treatment time, if we increase the radiation dosage *γ* and decrease the treatment time *L* at the same time, the cancer will also win the competition.

Throughout Figures 1–12, we choose the initial values *x*_1_(0) = 0.5 ⩽ *K*_1_ and *x*_2_(0) = 0.8 ⩽ *K*_2_. Also note that the choice of the values in the theorems are based on the range determined by [3], but they do not come from any real cell populations.

## 6. Conclusion and Discussion

In this paper, we employed a pair of ordinary differential equations to model the dynamics between the healthy cells and cancer cells for the cancer treatment by radiotherapy. We separated the treatment into two stages: treatment stage and recovery stage (no treatment stage). During the treatment stage, the radiation harvesting amount is *γx*_2_ to the cancer cells and *ɛγ* *x*_1_ to the healthy cells. However, during the recovery stage, the model has taken the most basic Lotka-Volterra competition type. We gave the range of radiation dosage *γ* under the following three results: the healthy cells and cancer cells are coexist, the cancer eradication periodic solution is globally stable, and the cancer win periodic solution is globally stable. Note that, in the discussion on the global stability of the cancer eradication periodic solution, we showed the relationship between the radiation dosage *γ* and the treatment time *L*; that is, *ɛγL* < *α*_1_ *ω* and *γ*(*ω* − *L*) < *ηω*, which are also given during the discussion on the cancer win periodic solution (*γL* < *α*_2_ *ω*). However, during the discussion on the coexistence of the healthy cells and cancer cells, we only give the range of the radiation dosage *γ*, which shows that it fits all the treatment time *L* ∈ (0, *ω*). This is a flaw, which is caused by the analysis methods in this paper. We will improve our analysis techniques and make a further study in the future.

The cancer treatment model discussed in this paper is only based on one treatment measure. It may be more effective for the cancer treatment if we add a medication during the recovery stage, which is still an open problem and we will carry out the research in the further work.
